# Structure–Function Relationships in High-Moisture Meat Analogues: Effects of Soybean Residue (Okara) on Plant Protein–Starch Gels

**DOI:** 10.3390/gels11100805

**Published:** 2025-10-07

**Authors:** Aunchalee Aussanasuwannakul, Thidarat Pantoa, Worapol Pengpinit

**Affiliations:** 1 Department of Food Chemistry and Physics, Institute of Food Research and Product Development, Kasetsart University, Bangkok 10903, Thailand; 2 Department of Manufacturing and Distribution, Institute of Food Research and Product Development, Kasetsart University, Bangkok 10903, Thailand

**Keywords:** okara, high-moisture extrusion, meat analogues, soy protein isolate, wheat gluten, protein–fiber interactions, texture analysis, SDS-PAGE, rheology, sustainable food systems

## Abstract

Okara, a fiber-rich soybean byproduct, can improve the sustainability of plant-based meats but may compromise texture when used at high levels. This study investigated the effects of okara flour (0–40%) on the structure–function relationships of high-moisture meat analogues (HMMA) formulated with soy protein isolate, wheat gluten, and corn starch. Analyses included composition, macrostructure, instrumental texture (cutting tests and TPA, evaluated by PCA), SDS-PAGE, and pasting behavior under both pressurized and atmospheric conditions. Increasing okara decreased protein density but increased fiber and fat, resulting in nutritional trade-offs. Fibrous anisotropy was preserved up to 20% okara but declined at higher levels, producing dense, isotropic matrices. Texture analyses revealed reduced firmness, cohesiveness, and elasticity, consistent with SDS-PAGE evidence of diminished 7S and 11S subunits. Rheological tests indicated suppressed starch swelling yet greater viscosity stability under pressure. Overall, moderate okara incorporation increased dietary fiber without fully compromising texture, whereas higher levels disrupted protein alignment and gel functionality.

## 1. Introduction

The growing demand for sustainable protein sources has accelerated the development of plant-based meat analogues (PBMAs) as alternatives to conventional meat products [1,2]. High-moisture extrusion (HME) is the leading technology for producing fibrous, meat-like structures because of its scalability and capacity to replicate anisotropic textures [3,4,5]. In this context, anisotropy refers to directional differences in structure, as seen in real meat where aligned muscle fibers cause texture to vary along versus across the grain [6]. HME replicates this fibrous, direction-dependent structure by subjecting hydrated proteins to heat, pressure, and shear, then stabilizing the alignment through cooling dies [7,8,9]. Although HME has been applied to diverse protein and starch sources, achieving meat-like texture remains challenging when fiber-rich ingredients are introduced, as they alter protein interactions and water distribution [10].

Soy protein isolate (SPI) is widely used in PBMAs for its high protein content and gel-forming ability [11,12]. Its functionality depends largely on the ratio of β-conglycinin (7S) to glycinin (11S), globulins with distinct solubility and gelation properties: higher 7S/11S ratios favor elasticity, whereas 11S-rich fractions promote hardness and ordered fibrous structures [13,14]. Prior studies show that the 7S/11S ratio strongly affects textural qualities. A higher proportion of 7S yields a more elastic, deformable network, while glycinin-rich systems form harder, more rigid gels due to cysteine-rich subunits that create extensive disulfide cross-links [15]. In contrast, β-conglycinin lacks cysteine in one major subunit, limiting cross-link density and increasing elasticity [15]. During extrusion, proteins aggregate into high-molecular-weight complexes while partially dissociating into smaller peptides, both processes contributing to network formation [16,17]. Protein–protein and protein–starch compatibilities further affect fibrous development: wheat gluten enhances alignment through its viscoelasticity, while starches act as fillers or structural modifiers depending on their gelatinization and retrogradation behavior [18]. However, incompatibility between SPI and gluten has also been reported, producing two-phase gels stabilized by covalent and noncovalent interactions [19].

Wheat gluten was included as a co-protein in our formulation to leverage its well-known structuring ability in HME. Its glutenin–gliadin network provides viscoelasticity and forms new disulfide linkages during extrusion, strengthening and aligning the gel matrix [20]. Although unsuitable for gluten-intolerant consumers, gluten remains widely used in meat analogues due to its unmatched capacity to improve fibrous texture [20].

Rheological studies further emphasize the roles of protein solubility and fiber in pasting and gelation. Soluble proteins such as SPI produce pronounced viscosity peaks during gelatinization, whereas fiber-rich ingredients such as hemp protein and brewer’s spent grain suppress starch swelling and reduce peak viscosity [21,22]. Moderate fiber levels can stabilize viscosity and improve hydration, but excessive fiber disrupts swelling and alignment, leading to softer, isotropic matrices lacking fibrous orientation [10,23,24,25]. These divergent outcomes highlight the delicate balance between nutritional enrichment and structural integrity in PBMA formulation.

Okara, the insoluble soybean residue from soymilk and tofu production, represents a promising yet underutilized byproduct in this context. It is rich in dietary fiber, residual protein, and lipids, but is still largely discarded or relegated to low-value animal feed [26,27]. Incorporation into bakery, snack, and noodle products increases fiber but often reduces elasticity, hydration, and structural integrity at higher levels [28,29]. Studies on bread, steamed bread, and noodles show that adding 10–25% okara substantially increases dietary fiber (~7% TDF in final products) while modifying dough properties [30,31,32,33]. In PBMAs, okara offers nutritional and sustainability benefits, yet its high fiber content may dilute protein concentration, interfere with network formation, and alter water redistribution during extrusion [10,14]. To date, systematic studies of okara in SPI–gluten–starch matrices under HME are limited. Okara has also been explored as a source of soluble soybean polysaccharides (SSPS), which serve as stabilizers and emulsifiers in foods and other applications [34,35,36,37].

This study addresses this gap by evaluating the effects of okara flour incorporation (0–40%) on the structure–function relationships of high-moisture meat analogues (HMMA). A multiscale approach was applied, including compositional analysis, macrostructural morphology, instrumental texture with multivariate analysis, protein subunit distribution by SDS-PAGE, and pasting behavior under pressurized and atmospheric conditions. By linking compositional shifts to molecular interactions, rheology, and texture, the work provides mechanistic insights into how okara modulates fibrous integrity. Findings show that moderate okara incorporation can increase dietary fiber without fully compromising texture, whereas higher levels disrupt protein alignment and anisotropy. These results offer practical guidance for designing sustainable, fiber-enriched meat analogues.

## 2. Results and Discussion

### 2.1. Chemical Composition

Dietary fiber increased markedly with okara addition, from 0.00 g/100 g in the control to 7.59 g/100 g at 40% Okara. By comparison, the defatted soy flour (DSF) reference contained only 1.58 g/100 g fiber despite its high protein content (24.63%). Energy ranged from 161.57 to 210.98 kcal/100 g, with energy from fat rising from 14.89 to 46.33 kcal/100 g as okara content increased (Table 1).

These compositional shifts highlight the nutritional trade-offs of okara substitution. Protein dilution was expected and is consistent with findings on other fiber-rich byproducts such as faba bean press cake [22] and brewer’s spent grain [25]. The increase in fat, while lowering the protein-to-energy ratio, may improve juiciness and mouthfeel in plant-based analogues [14]. Fiber enrichment represents the most notable benefit, aligning with [10], who reported that added fiber increases dietary fiber while altering hydration properties. Similarly, studies on cereal-based products incorporating 10–25% okara showed increased dietary fiber (~7% TDF) but reduced elasticity, hydration, and sensory acceptability at higher levels [30,32,33]. The rise in moisture at moderate okara levels reflects fiber’s water-binding capacity, whereas the decline at 40% incorporation suggests excessive fiber disrupts protein–starch interactions, similar to other high-fiber formulations [38]. Ash remained relatively stable, confirming that mineral input from okara was minor, while carbohydrate variation largely reflected shifts among other macronutrients [17].

Overall, okara substitution decreased protein density but enhanced fiber and lipid contributions, altering both nutritional quality and water distribution. These changes provide a mechanistic basis for subsequent differences in pasting, gelation, and texture, underscoring okara’s dual role as a functional fiber source and a modulator of HMMA structure.

### 2.2. Macrostructure and Fibrous Integrity

**Figure 1:**
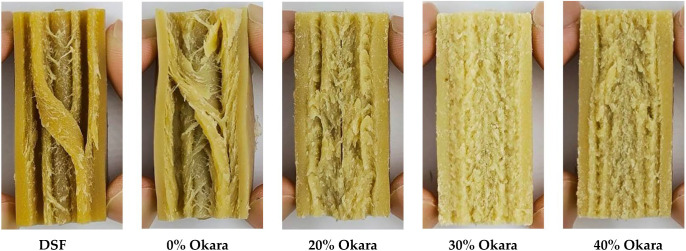
Longitudinal tear of high-moisture meat analogue (HMMA) samples along the extrusion direction, showing fibrous alignment. From left to right: 100% defatted soy flour (DSF), control (0% okara), and formulations with 20%, 30%, and 40% okara. DSF and control samples exhibit clear lamellar fibers, while increasing okara reduces fiber definition and leads to denser, more isotropic structures.

The internal macrostructure of HMMA samples torn longitudinally is shown in Figure 1. The DSF reference exhibited well-defined lamellar fibers with deep fissures and parallel bundles, yielding a strongly anisotropic, meat-like structure. The control (0% okara) also formed aligned fibrous strands, though slightly more compact and less defined.

At 20% Okara incorporation, fibrous orientation remained evident but appeared irregular, with wavy layers and compression zones indicating partial disruption of lamellar continuity. At higher incorporation levels, fibrous architecture deteriorated progressively: the 30% sample displayed a spongy morphology, likely due to intermediate levels of fiber disrupting lamellar continuity without fully collapsing the matrix. Both 30% and 40% samples appeared broadly isotropic, although the 40% formulation showed excessive fiber compaction that suppressed expansion, yielding a dense, isotropic network with little directional orientation. Thus, anisotropic structuring was largely retained up to ~20% Okara but diminished beyond this threshold.

These morphological changes reflect the compositional shifts described in Section 2.1. Reduced protein availability at higher okara levels may have limited the building blocks for fiber formation, restricting alignment and elongation during extrusion. Insoluble fiber likely introduced steric hindrance that interrupted protein phase separation, while residual lipids may have reduced melt flowability and hindered alignment. Comparable mechanisms have been reported in other HME studies, where excessive fiber disrupts fibrous structuring [22,25], altered protein composition reduces anisotropy [14], and fiber incorporation affects hydration and matrix compactness [10].

In summary, anisotropy was retained at 20% Okara but progressively lost at higher levels. While levels below 20% were not tested here, future work will examine intermediate incorporations (e.g., 10%) to refine this boundary. This link between composition and macrostructure emphasizes the need to balance protein concentration with fiber enrichment when designing sustainable, meat-like analogues.

### 2.3. Instrumental Texture Characterization Using Multivariate Analysis

In addition to PCA, the complete results of cutting and TPA measurements are provided in Supplementary Table S1 (*n* = 5 for cutting tests; *n* = 10 for TPA), showing mean ± SD values for each parameter across samples.

#### 2.3.1. PCA of Cutting Test Variables

To address multicollinearity among the nine cutting variables, correlation screening was first performed (Appendix A, Table A1). Highly correlated measures (r > 0.95) were reduced to a representative set: p.Work_of_Shear, p.Cutting_Strength, v.Force, v.Distance_at_Failure, and Texturization. PCA of these five dimensions explained 82.79% of the variance (PC1 = 52.84%, PC2 = 29.94%). PC1 reflected overall cutting resistance (p.Work_of_Shear, v.Force), while PC2 represented structural anisotropy and failure behavior (p.Cutting_Strength, Texturization).

**Figure 2:**
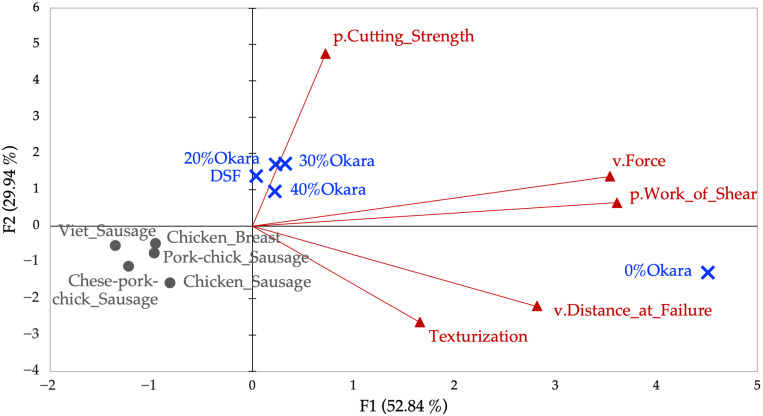
Principal component analysis (PCA) biplot of cutting test texture variables across prototype and commercial samples. The first two principal components (PC1 = 52.84%, PC2 = 29.94%) jointly explain 82.79% of the variance. Active variables (red arrows/triangles) represent loadings for p.Work_of_Shear, p.Cutting_Strength, v.Force, v.Distance_at_Failure, and Texturization. Points represent individual samples. 0% Okara is positioned at the far right with high PC1 scores, reflecting strong firmness and cutting resistance. Commercial sausages cluster in the lower-left quadrant, indicative of soft texture and high deformability. 20–40% Okara and Defatted Soy Flour (DSF) cluster along PC2, reflecting moderate firmness but higher internal cohesion.

Sample distribution revealed clear contrasts (Figure 2). 0% Okara scored highest on PC1, indicating firm, fibrous texture. 20–40% Okara clustered with DSF along PC2, reflecting moderate firmness but greater cohesion, while commercial sausages grouped in the lower-left quadrant, showing soft, deformable textures. Chicken breast aligned separately, consistent with its fibrous tearing behavior.

#### 2.3.2. PCA of TPA Variables

Similarly, correlation screening of the eight original TPA parameters (Appendix A, Table A2) identified strong collinearity among Force, Hardness, Gumminess, and Chewiness (r > 0.98). Four representative variables—Chewiness, Cohesiveness, Springiness, and Adhesiveness—were retained. PCA of these parameters explained 86.47% of the variance (PC1 = 50.40%, PC2 = 36.07%). PC1 captured a gradient from chewy to adhesive textures, while PC2 represented elasticity and recovery (Cohesiveness, Springiness).

**Figure 3:**
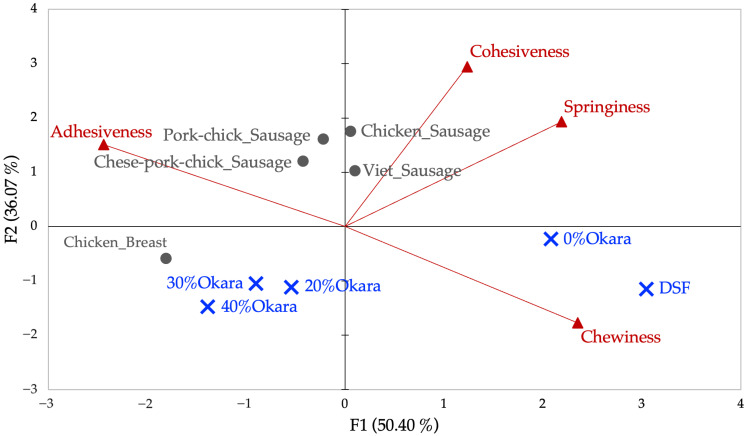
Principal component analysis (PCA) biplot of reduced texture profile analysis (TPA) parameters. Four variables (Chewiness, Cohesiveness, Springiness, and Adhesiveness) were retained after correlation screening. The first two principal components (F1 = 50.40%, F2 = 36.07%) explain 86.47% of the total variance. Arrows represent variable loadings; points represent sample scores. Defatted soy flour (DSF) and 0% Okara are positioned on the far right with high chewiness and low adhesiveness, while higher okara samples (20–40% Okara) shift leftward toward softer, more adhesive textures. Meat-based sausages cluster in the upper quadrants, reflecting higher cohesiveness and springiness.

As shown in Figure 3, DSF and 0% Okara plotted to the far right with high chewiness and low adhesiveness, while 20–40% Okara shifted leftward, reflecting softer, more adhesive textures. Among animal products, sausages clustered in the upper quadrants, associated with cohesiveness and springiness, whereas chicken breast occupied a lower position, reflecting fibrous but less cohesive structure.

Taken together, PCA of cutting and TPA datasets demonstrates that texture is multidimensional. Cutting reflects resistance and anisotropy, while TPA captures elasticity and oral deformation. 0% Okara aligned with dense, fibrous traits, while higher okara incorporations yielded softer, adhesive, less cohesive textures. DSF was firm but brittle, while commercial sausages showed low cutting resistance but high elasticity, reflecting emulsified matrix systems.

These outcomes align with earlier findings: higher protein promotes fibrous structuring [39], whereas elevated fiber and fat soften matrices and reduce anisotropy [10,22,25]. Notably, differences in extrusion parameters may explain why [22,25] observed stronger anisotropy at moderate fiber incorporation, whereas our shear-driven, lower-temperature process emphasized cohesion at the expense of elasticity.

The PCA framework confirms that moderate okara incorporation maintains some fibrous characteristics but progressively compromises chewiness, springiness, and anisotropy at higher levels. While this enhances sustainability and dietary fiber, compensatory strategies (e.g., protein blending or binders) are needed to restore meat-like bite. These mechanical findings provide a macroscopic view of how formulation shifts affect texture, leading to the next question: how does okara alter protein subunit distribution and cross-linking at the molecular level? This was examined through SDS-PAGE (Section 2.4).

### 2.4. Protein-Level Characterization Using SDS-PAGE

**Figure 4:**
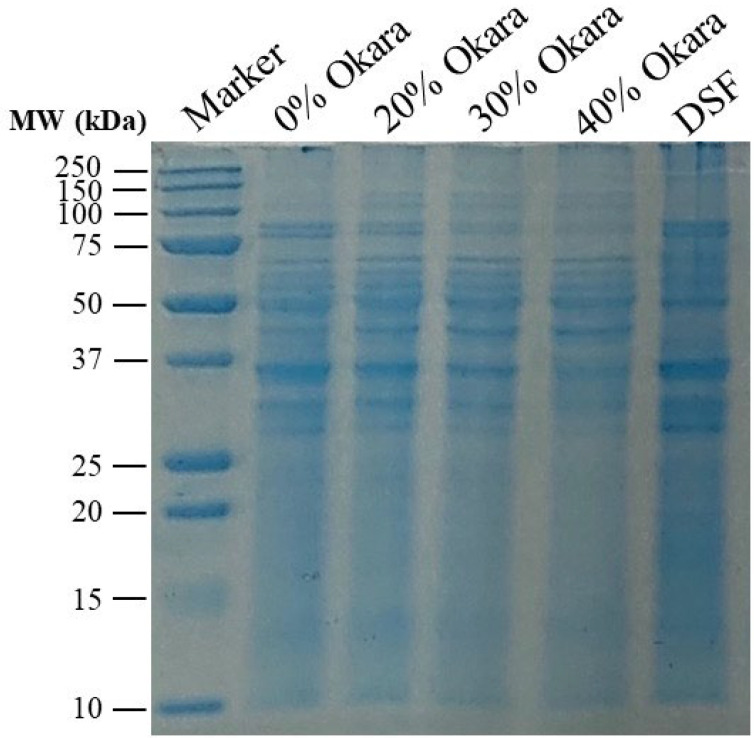
SDS–PAGE profiles of okara-based HMMA (0–40% okara) compared to defatted soy flour (DSF). Major soybean protein subunits (β-conglycinin α′, α, β; glycinin acidic and basic) are visible in the control and DSF lanes but progressively weaken with higher okara levels. The fading bands reflect reduced protein purity, stronger aggregation, and fiber masking effects at high okara substitution.

The SDS-PAGE profiles of HMMA formulations with increasing okara levels (0–40%) are shown in Figure 4, with defatted soy flour (DSF) as reference. In the control (0% okara), distinct soybean storage proteins were visible: β-conglycinin (7S; α′ ~72 kDa, α ~68 kDa, β ~50 kDa) and glycinin (11S; acidic ~35 kDa, basic ~20 kDa) subunits. This profile closely resembled DSF, indicating the dominance of SPI-derived proteins.

With higher okara incorporation, band intensity decreased progressively. At 20% okara, β-conglycinin bands were weaker, while at 30% the α′ and α bands were faint and acidic glycinin was further diminished. At 40% okara, only traces of β (~50 kDa) and basic glycinin (~20 kDa) remained. These lighter profiles reflect reduced protein purity and solubility compared with DSF.

The fading of 7S and 11S subunits with increasing okara suggests stronger aggregation and reduced solubility during extrusion. Similar patterns have been reported under high extrusion intensity, where vicilin and legumin bands disappear due to high-molecular-weight aggregation [13,16,40]. In our case, the loss of α′/α and acidic glycinin subunits indicates less available soluble protein to align into fibrous networks.

Okara’s fiber further contributes to weaker bands. Psyllium husk, for example, has been shown to reduce visibility of pea protein subunits through solubility masking [41], while [42] linked lower solubility to more compact fibrous matrices. Thus, okara’s high fiber and lower protein density are consistent with both greater aggregation and matrix interference, which can diminish apparent band intensity; similar trends have been reported for fiber-rich systems.

These molecular changes are consistent with the structural observations: extrudates with 30–40% okara exhibited compact, isotropic morphologies and softer textures, reflecting fewer soluble subunits for anisotropic alignment. Prior work highlights that balanced retention of soluble subunits is critical for structuring [17] and that higher 7S/11S ratios improve fibrous texture [14]. Replacing SPI with okara reduced both protein availability and effective 7S/11S ratios, while fiber promoted insolubility, together weakening texturization.

From a nutritional perspective, aggregation does not necessarily hinder digestibility. Ref. [25] reported that plant-based HMMAs digest more rapidly than chicken, with extrusion-modified proteins breaking down into ~10 kDa peptides. Thus, okara proteins, though less soluble in SDS-PAGE, likely remain enzymatically accessible, although the fiber matrix may slow release.

In summary, SDS-PAGE confirms that increasing okara substitution reduces the visibility of 7S and 11S subunits through aggregation, lower protein purity, and fiber masking. These molecular-level changes explain the loss of fibrous integrity and softer textures in high-okara formulations, reinforcing the composition–structure–function relationships observed across this study.

### 2.5. Pasting Properties of HMMA Flour Mixtures

#### 2.5.1. Gelling Behavior Under Pressure

**Figure 5:**
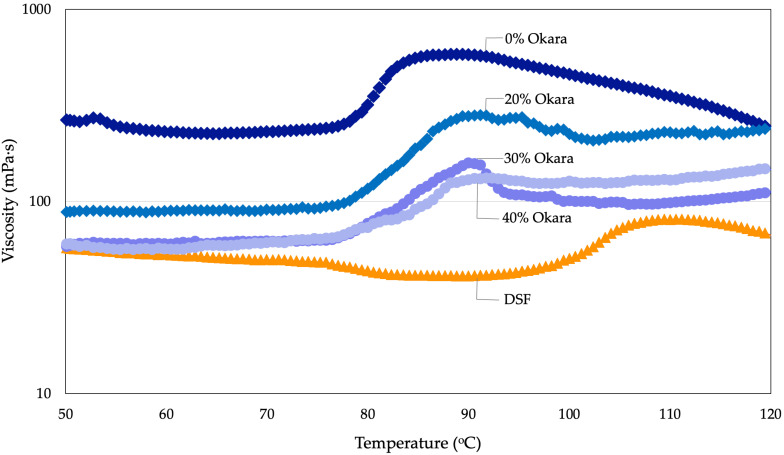
Viscosity–temperature profiles of HMMA flour mixes (0–40% Okara) and defatted soy flour obtained under pressurized conditions (30 bar, 50–120 °C) at a constant shear rate of 200 s^−1^; apparent viscosity (mPa·s). The starch-rich 0% Okara mix showed the most pronounced gelatinization peak, while okara-containing blends exhibited progressively flatter, more stable curves. DSF showed minimal viscosity development due to its low starch content.

Under pressurized conditions (30 bar, 50–120 °C), pasting temperatures of HMMA flour mixes ranged from 76.4 to 78.3 °C, markedly lower than defatted soy flour (97.3 °C) (Table 2). The starch-rich 0% Okara mix displayed the highest peak viscosity (583.8 mPa·s) and final viscosity (228.5 mPa·s), while viscosity decreased sharply with okara incorporation, reaching 132.5 mPa·s at 40% Okara. Viscosity–temperature curves (Figure 5) confirmed these patterns: the control showed a pronounced gelatinization peak, whereas okara blends produced flatter, more stable profiles. Measurements were performed at a constant shear rate of 200 s^−1^ in the PR30/ST pressure cell, and apparent viscosity was expressed in mPa·s. DSF showed minimal viscosity development, reflecting its low starch content.

Under pressurized conditions, the markedly higher pasting temperature of DSF (97.3 °C) compared with the starch-containing formulations likely reflects its very low starch fraction and predominance of protein, which resists hydration and gelatinization. In contrast, starch-rich samples (0% okara) displayed pronounced gelatinization peaks with high peak viscosity. Incorporation of okara progressively suppressed viscosity, consistent with insoluble dietary fiber competing for water and physically hindering starch granule swelling. At higher levels (30–40%), this fiber interference dominated, resulting in dramatically flattened curves and much lower apparent viscosities.

#### 2.5.2. Pasting Properties Under Atmospheric Conditions

**Figure 6:**
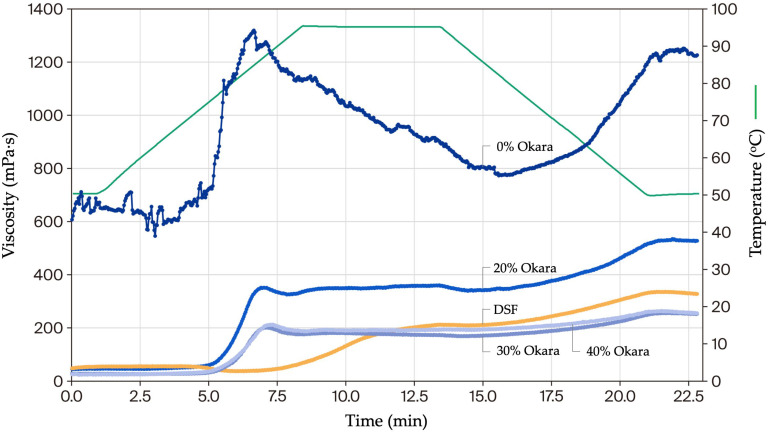
Viscosity–time profiles of HMMA flour mixes (0–40% Okara) and defatted soy flour from the RVA-like atmospheric test (50–95–50 °C). The 0% Okara control displayed a sharp peak and setback, whereas increasing okara incorporation dampened swelling and retrogradation. DSF produced weak viscosity development characteristic of protein-dominated systems.

In the RVA-like test (50–95–50 °C), defatted soy flour showed the lowest pasting temperature (53.2 °C), while okara blends peaked around 69–71 °C (Table 2). The 0% Okara control exhibited exceptionally high viscosity (peak 1318.3 mPa·s; final 1209.7 mPa·s), more than sixfold higher than the 40% Okara mix (~212–257 mPa·s). Okara-containing samples displayed progressively dampened swelling and reduced setback, while DSF produced only moderate viscosity (327.6 mPa·s). These viscosity–time curves (Figure 6) highlighted classic starch-driven swelling and retrogradation in the control, which were attenuated as fiber levels increased.

The pasting data reveal complementary roles of starch and okara-derived fiber in shaping HMMA rheology. Starch-rich systems (0% Okara) generated high swelling and setback but were unstable under heat, consistent with breakdown during gelatinization. Okara incorporation suppressed peak viscosity and setback while flattening the curves, indicating competition for water and steric hindrance of starch swelling. At 40% Okara, this competition was so strong that amylose leaching and reassociation during cooling were largely prevented, resulting in the absence of a setback peak. DSF showed minimal viscosity development, reflecting its low starch content, and its very high protein fraction further delayed the onset of viscosity increase. This resistance to hydration and gelatinization explains the substantially higher pasting temperature observed for DSF compared with starch-containing blends. Although protein–fiber associations may also contribute to viscosity stabilization, the predominant mechanism for reduced viscosity appears to be suppression of starch swelling by insoluble fiber, which limits granule expansion and amylose leaching.

At the same time, insoluble fiber likely promoted protein–fiber associations that stabilized viscosity; additionally, interactions with leached amylose during gelatinization/cooling may contribute to network thickening, albeit to a lesser extent than in the starch-richer control [21,22]. Beyond its insoluble fraction, okara also contains pectic polysaccharides, which may contribute to water-binding, viscosity stabilization, and potential prebiotic effects in functional foods [37,43,44,45,46,47].

These rheological outcomes integrate with earlier analyses. SDS-PAGE showed reduced solubility of 7S/11S subunits at high okara levels, while macrostructural and textural tests confirmed loss of fibrous alignment and elasticity. The attenuated viscosity peaks observed here represent the mechanistic link: limited starch swelling combined with aggregated proteins produces compact, isotropic textures. Similar stabilization by fiber–protein interactions has been reported in lentil and pea formulations under high-pressure RVA [10].

Atmospheric RVA profiles further underscored this effect. The 0% Okara control mirrored its firm, brittle texture by exhibiting strong swelling, breakdown, and setback, whereas okara blends paralleled their softer, more hydrated textures through dampened transitions. DSF’s weak viscosity development was dominated by protein aggregation, in line with earlier observations of low-solubility proteins [42].

In summary, okara acts both as a diluent of starch swelling and a stabilizer of protein–fiber networks. Moderate incorporation preserves some anisotropy, but higher levels (30–40% Okara) yield dense, isotropic textures. Pasting analysis thus provides the rheological bridge connecting compositional shifts to molecular aggregation and macroscopic texture, reinforcing the structure–function framework of this study.

## 3. Conclusions

This study demonstrated how okara incorporation (0–40% Okara) modulates the structure–function relationships of high-moisture meat analogues (HMMA). Increasing okara reduced protein density but enriched fiber and fat, producing nutritional gains alongside structural trade-offs. Fibrous anisotropy was retained around 20% Okara but deteriorated at higher levels, leading to dense, isotropic textures.

Instrumental analyses confirmed weaker fibrous resistance, elasticity, and cohesiveness in okara-rich samples, while SDS-PAGE and pasting tests revealed reduced protein solubility and suppressed starch swelling as underlying mechanisms. Together, these results show that moderate okara incorporation can enhance dietary fiber without fully compromising texture, whereas higher levels disrupt protein alignment and gel functionality.

Overall, okara represents a sustainable, fiber-rich ingredient for plant-based meats, but successful application requires complementary formulation strategies (e.g., protein blending, binders, or texturizers) to balance nutritional enrichment with desirable meat-like texture. Future work will examine < 20% incorporations (e.g., 10%) to refine the anisotropy boundary.

## 4. Materials and Methods

### 4.1. Materials

Okara flour was obtained from fresh soybean residue (okara) supplied by a local tofu and soymilk producer (Ngow Jeng Ngoun Co., Ltd., Bangkok, Thailand). The okara was tray-dried at 60 °C until the moisture content was reduced to below 10%, then ground and passed through a 100-micron mesh sieve to obtain a uniform powder. The resulting okara flour used in this study contained 4.33% moisture, 29.35% protein, 12.85% fat, 1.28% soluble dietary fiber (SDF), and 36.93% insoluble dietary fiber (IDF), yielding a total dietary fiber (TDF) content of 38.21% (as-is basis) [48]. Soy protein isolate (≥90% protein, ~1% fat) and vital wheat gluten (~80–85% protein, ~2% fat) were food-grade ingredients obtained from Bangkok Chemical Co., Ltd., Bangkok, Thailand. Corn starch (≥98% carbohydrate, ~0.3% protein, ~0.1% fat) was sourced from the same supplier. Defatted soy flour (DSF; Omshree Agro Tech Pvt. Ltd., Dhule, India) contained 52.51% protein, 7.89% moisture, 0.89% fat, 3.4% fiber, and 4.98% ash and was used as a reference sample prepared entirely from DSF (hereafter referred to as “DSF” in tables and figure captions for consistency with the okara incorporation levels). The formulation of raw materials for producing high-moisture meat analogues (HMMA) and the chemical composition of the produced HMMA are shown in Table 1. A gluten–starch-only control was not tested, as preliminary trials indicated it did not yield coherent extrudates under high-moisture extrusion (Table 3).

Commercial products used as texture benchmarks included the following: Commercial 1: Tender Chicken Breast by CPF (Thailand) PCL, Bangkok, Thailand; chicken breast meat 83%. Commercial 2: Vietnamese Sausage by S. Khonkaen Food PCL, Samut Prakan, Thailand; pork 72% and chicken 8%. Commercial 3: Chicken Frank Sausage by CPF Food and Beverage Co., Ltd., Bangkok, Thailand; chicken breast meat 65%. Commercial 4: Smoky Bite (Pork and Chicken Sausage), Ezy Taste Brand, CPF Food and Beverage Co., Ltd., Bangkok, Thailand; pork 60% and chicken 10%. Commercial 5: Cheese Bite (Pork and Chicken Sausage), Ezy Taste Brand, CPF Food & Beverage Co., Ltd., Bangkok, Thailand; pork 50%, chicken 19%, and cheese 10%.

All chemicals and reagents used in analyses were of analytical grade. Cholesterol (≥99% purity, Sigma-Aldrich, St. Louis, MO, USA), D-(+)-glucose (≥99.5% purity, Sigma-Aldrich, St. Louis, MO, USA), and 10% (*v*/*v*) trichloroacetic acid (Sigma-Aldrich, St. Louis, MO, USA) were used as received. The cholesterol content in the supernatant was determined using a Total Cholesterol Assay Kit (Colorimetric) (Cell Biolabs, Inc., San Diego, CA, USA), while glucose content was measured by a Glucose Oxidase Assay Kit (Fluorometric) (Abcam, Cambridge, UK). For SDS-PAGE, Laemmli Sample Buffer containing 5% 2-mercaptoethanol (Bio-Rad Laboratories, Hercules, CA, USA) and protein molecular weight markers (10–200 kDa, Precision Plus Protein All Blue Standards™, Bio-Rad Laboratories, Hercules, CA, USA) were used.

### 4.2. Preparation of High-Moisture Meat Analogue (HMMA)

HMMAs were prepared using an intermeshing co-rotating twin-screw extruder (Hermann Berstorff Laboratory, ZE25 × 33D, Hannover, Germany) with a length-to-diameter (L/D) ratio of 33:1. The extruder comprised seven barrel sections and was equipped with a long square-shaped cooling die (2.0 cm width × 0.5 cm height). The maximum barrel temperature was set at 120 °C at barrel zone 6, with die pressure maintained at ~25–30 bar and mean residence time of ~30–40 s. The feed moisture content was adjusted to 55%, and the screw speed was maintained at 350 rpm. The feed rate was 2.88 kg/h.

After reaching steady-state conditions, extrudates were continuously collected at the end of the cooling die. Samples were immediately sealed in laminated plastic bags and stored at 4 °C. Texture profile analysis (TPA) was conducted after ~18 h of storage at 4 °C. For compositional and functional property analyses, portions of the extrudates were stored at −18 °C.

### 4.3. Proximate Composition and Nutritional Value

The proximate composition of the samples was determined using standard AOAC methods: moisture (AOAC 934.01), protein (AOAC 990.03), fat (AOAC 920.39), ash (AOAC 942.05), carbohydrate (by difference), dietary fiber (AOAC 985.29, 991.42, 993.19), and total energy and energy from fat (calculated by Atwater factors). The overall analytical framework followed previously described protocols [29,49]. The contents of total dietary fiber (TDF) were assayed using the enzymatic–gravimetric method (AOAC 985.29, AOAC 991.42, and AOAC 993.19), as described by [50].

### 4.4. Functional Properties of HMMA Samples

#### 4.4.1. Sample Preparation

HMMA samples prepared as described in Section 4.2 were stored at −18 °C and thawed at 4 °C prior to analysis. After thawing, samples were reduced to small pieces to facilitate handling. For CAC, GAC, and SDS-PAGE, samples were used directly in their fresh (wet) form. When size reduction was required, samples were ground to pass through a 60-mesh sieve before analysis.

#### 4.4.2. Cholesterol Adsorption Capacity (CAC)

The 1 mg/mL cholesterol solution (see Section 4.1 for reagent details) was prepared in ethanol. The pH of the solution was adjusted to 2.0 with 1 M HCl or 7.0 with 1 M NaOH, simulating gastric and intestinal environments, respectively. These pH levels were chosen to reflect the conditions that the HMMA might encounter during digestion, allowing for an understanding of how okara flour affects cholesterol adsorption under physiologically relevant conditions. Next, 2.0 g of HMMA sample was dispersed in 100 mL of cholesterol solution by continuous stirring at 37 °C for 2 h followed by centrifugation at 6000× *g* for 15 min. The cholesterol content in the supernatant was determined using a Total Cholesterol Assay Kit (Cell Biolabs, Inc., San Diego, CA, USA). The cholesterol adsorption capacity was calculated using the following equation:

$$CAC\left(mg/g\right)=\;\frac{Cholesterol\;before\;adsorption\;(mg)-Cholesterol\;after\;adsorption\;(mg)}{Weight\;of\;dry\;sample\;(g)}$$

#### 4.4.3. Glucose Adsorption Capacity (GAC)

One gram of HMMA sample was dispersed in 100 mL of 50 mM glucose solution (see Section 4.1 for reagent details) and stirred continuously at 37 °C for 6 h followed by centrifugation at 4000× *g* for 20 min. The glucose content in the supernatant was measured by Glucose Oxidase Assay Kit (Abcam, Cambridge, UK). The glucose adsorption capacity was calculated using the following equation:

$$GAC\left(mM/g\right)=\;\frac{Glucose\;before\;adsorption(mM)-Glucose\;after\;adsorption\;(mM)}{Weight\;of\;dry\;sample\;(g)}$$

### 4.5. SDS PAGE

Samples were characterized by sodium dodecyl sulfate–polyacrylamide gel electrophoresis (SDS–PAGE). HMMA samples were weighed (500 ± 10 mg) and extracted with 500 mL of 50 mM Tris–HCl buffer containing 50 mM dithiothreitol (DTT), 7 M urea, 2 M thiourea, and 2% CHAPS (3-[(3-cholamidopropyl)dimethylammonio]-1-propanesulfonate), pH 8.8. The solution was sonicated at 60 °C for 5 min (three repeats) and then centrifuged (14,000× *g*) at 4 °C for 30 min. The supernatant (50 μL) was mixed with 50 μL of Laemmli Sample Buffer containing 5% 2-mercaptoethanol before heating to 100 °C for 5 min. Samples, along with protein markers (see Section 4.1), were loaded onto a 12% acrylamide separating gel overlaid with a 4% stacking gel. Electrophoresis was performed at 200 V for 40 min. The gel was fixed in 50% (*v/v*) methanol and 10% (*v/v*) trichloroacetic acid for 2 h, then washed three times with distilled water and stained with Coomassie Brilliant Blue R-250 (Thermo Fisher Scientific, Waltham, MA, USA) for 15 min before destaining overnight with continuous shaking.

### 4.6. Rheometer-Based Pasting Properties of HMMA Flour Mixtures

#### 4.6.1. Determination of Gelling Behavior Under Pressure

**Figure 7:**
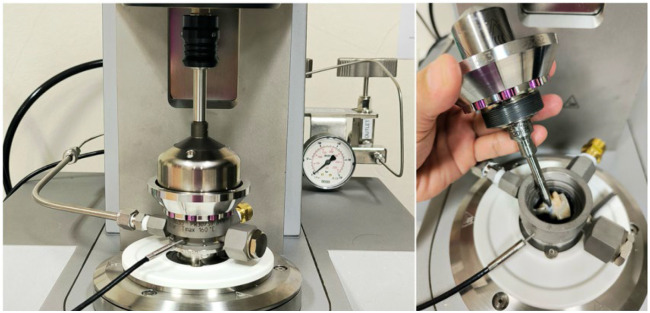
Anton Paar PR30/ST starch pressure cell (fitted with Pt-100 temperature probe) used for determination of gelling behavior under pressure (Section 4.6.1). The closed, pressurized design allows simulation of high-moisture extrusion conditions at elevated temperature (up to 120 °C) and pressure (up to 30 bar).

The gelling behavior of DSF and HMMA flour mixes containing 0%, 20%, 30%, and 40% okara was determined using a controlled-stress rheometer (MCR 302 series, Anton Paar, Graz, Austria) equipped with a PR30/ST pressure starch cell fitted with a Pt-100 temperature probe (Figure 7). For each measurement, 1.96 g of flour was dispersed in 12.04 mL of deionized water to yield a total dispersion volume of 14 mL at a concentration of 0.14 g/mL. Dispersions were premixed with a magnetic stirrer until homogeneous and carefully loaded into the pressure cell, which was then sealed to prevent leakage under pressurization.

The measurement protocol simulated high-moisture extrusion barrel conditions through six sequential stages: (i) mixing at 50 °C and 0 bar with a stirring speed of 800 rpm for 30 s; (ii) hydration/equilibration at 50 °C and 0 bar at 200 rpm for 60 s; (iii) heating from 50 to 120 °C over 600 s under 30 bar at 200 rpm; (iv) holding at 120 °C and 30 bar for 600 s at 200 rpm; (v) cooling from 120 to 50 °C over 600 s at 0 bar and 200 rpm; and (vi) final holding at 50 °C and 0 bar for 120 s at 200 rpm. Apparent viscosity (η) was recorded every 2 s during isothermal stages (i, ii, v, vi) and every 5 s during heating and high-temperature holding (iii, iv) at a constant shear rate of 200 s^−1^ in the PR30/ST cell. Viscosity was calculated from instrument torque using RheoCompass™ software (Version 1.30, Anton Paar GmbH, Graz, Austria) and reported in mPa·s.

To provide a conventional reference alongside the extrusion-simulated test, an RVA-like pasting analysis was also conducted using the starch cell in pressureless mode.

#### 4.6.2. Determination of Pasting Properties (RVA-like Starch Cell Test)

**Figure 8:**
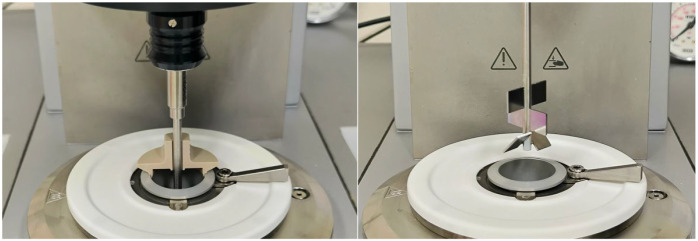
Anton Paar C-ETD 160/ST starch cell operated in pressureless mode for RVA-like pasting analysis (Section 4.6.2). The open system permits conventional heating–cooling cycles under atmospheric pressure, allowing comparison with standard starch pasting profiles.

For comparison, the pasting behavior of the HMMA flour mixtures was analyzed using the same rheometer equipped with a starch cell (C-ETD 160/ST, Anton Paar, Graz, Austria) operated in pressureless mode (Figure 8). Suspensions were prepared at the same concentration as in Section 4.6.1 directly in the starch cell canister. Powders were pre-wetted by stirring with a magnetic rod, and canister walls were carefully wiped to remove dry residues. After a short pre-shear at 960 rpm for 10 s, the impeller speed was set to 160 rpm for the remainder of the test. Data on torque (converted to apparent viscosity) and temperature were collected every 2 s.

The controlled temperature program consisted of five steps: (i) holding at 50 °C for 50 s to equilibrate; (ii) linear heating from 50 to 95 °C at 6 °C/min; (iii) holding at 95 °C for 5.0 min to assess paste stability; (iv) linear cooling from 95 to 50 °C at 6 °C/min; and (v) holding at 50 °C for 2.0 min to evaluate setback. Apparent viscosity was calculated from instrument torque by RheoCompass™ software (Version 1.30, Anton Paar GmbH, Graz, Austria) at a constant shear rate of 160 s^−1^ in the C-ETD 160/ST starch cell. Data were logged every 2 s and reported in mPa·s. From each curve, standard RVA-analog parameters were obtained, including pasting temperature (onset during heating), peak viscosity, and final viscosity (end of 50 °C hold). Each sample was tested in triplicate using freshly prepared dispersions. Between runs, the canister and impeller were thoroughly cleaned and dried, and temperature calibration and zero-gap checks were performed daily.

### 4.7. Texture Profile Analysis and Cutting Strengt

Texture profile analysis (TPA) and cutting strength tests were conducted using a texture analyzer (TA-XTplus^®^, Texture Technologies Corp., Scarsdale, NY, USA) equipped with a 50 kg load cell and operated via Texture Exponent software (version 3.0.5.0; Stable Micro Systems Ltd., Godalming, Surrey, UK).

For TPA, a cylindrical probe (50 mm diameter) was used in compression mode. Sample pieces (2 cm width × 0.5 cm height × 2 cm length) were equilibrated to room temperature prior to testing. The test was performed using the following settings: pre-test, test, and post-test speeds of 1 mm/s, 50% strain, and an auto-trigger force of 5 g. Each sample was tested in 10 replicates. The compression was performed under dry, non-lubricated conditions. Adhesion was not evident, as the HMMA samples had firm, cohesive surfaces that detached cleanly from the probe and platform after each cycle. The parameters obtained included force (g), hardness (g), adhesiveness (g·s), springiness, cohesiveness, gumminess, chewiness, and resilience. The following standard equations were used:

(1) $$\mathrm{Springiness}\;(\%)=\frac{D_{2}}{D_{1}}\times 100\;$$

$D_{1}$: distance at first maximum force; $D_{2}$: distance at second maximum force

(2) $$\mathrm{Cohesiveness}\;(\%)=\frac{A_{2}}{A_{1}}\times 100\;$$

$A_{1}$: area under the first compression curve; $A_{2}$: area under the second compression curve

(3) Chewiness (g)=Cohesiveness×Springiness×Peak Force 

(4) Gumminess (g)=Cohesiveness×Hardness 

Cutting strength analysis was performed using a blade set probe. Sample pieces (2 cm width × 0.5 cm height × 5 cm length) were cut in both the longitudinal (parallel, p) and transversal (v) directions relative to the extrusion flow. Settings: compression mode, 90% strain, 5 g auto-trigger force, and pre-/test-/post-test speeds of 1 mm/s. Each condition was repeated in more than five replicates. Cutting strength (CS) was calculated as:

(5) $${\mathrm{CS}\;(g/\mathrm{cm}}^{2})=\frac{\mathrm{Maximum}\;\mathrm{Force}\;(g)}{{\mathrm{Cross-sectional}\;\mathrm{Area}\;(\mathrm{cm}}^{2})}\;$$

The following parameters were collected for both directions: force, work of shear, cutting strength, and distance at failure. “Work of shear” represents the total energy required to cut through the sample and was calculated as the area under the force–distance curve, ∫F dx, by numerical integration in the texture analysis software. “Distance at failure” indicates blade displacement at fracture (sample extensibility).

**Figure 9:**
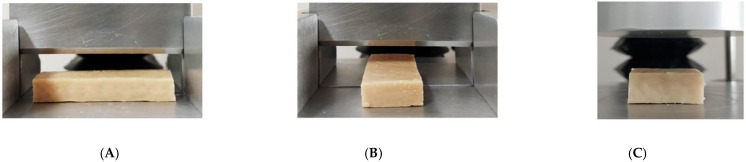
Experimental setups for texture analysis of HMMAs. (**A**) Longitudinal cutting (parallel to extrusion flow). (**B**) Transverse cutting (perpendicular to extrusion flow). (**C**) Texture profile analysis (TPA) using a cylindrical probe during double compression.

The degree of texturization was expressed as the ratio between force in the longitudinal direction and force in the transversal direction. A qualitative texturization score was also recorded based on the sample’s deformation pattern and resistance during cutting (Figure 9).

### 4.8. Data Processing and Statistical Analysis

Statistical analyses were performed using XLSTAT software (version 2025.1.2.1430; [51]) for Macintosh OS 15.6 (32-bit). One-way analysis of variance (ANOVA), followed by Tukey’s post hoc test, was used to compare the means of nutritional composition and functional property data among HMMA samples, as well as pasting property data among HMMA flour mixture samples. Results are expressed as mean ± standard deviation, with statistical significance set at *p* ≤ 0.05. All measurements were conducted in triplicate or more to ensure analytical reliability.

Principal component analysis (PCA) was conducted in XLSTAT to explore sample differentiation and reduce dimensional redundancy in the cutting test and TPA datasets. PCA was performed separately for each dataset using the correlation matrix to standardize variable scales. Redundant variables were identified based on high inter-variable correlations (Pearson’s r > 0.90), overlapping PCA loadings, and shared variance contributions. Representative variables were selected using the following criteria: absolute factor loadings (|loading| ≥ 0.70), squared cosines (cos^2^ ≥ 0.50), and contribution percentages to the first two principal components. For the cutting dataset, five variables were retained: Work of Shear (longitudinal), Cutting Strength (longitudinal), Force (transversal), Distance at Failure (transversal), and Texturization ratio. For the TPA dataset, four variables were retained: Chewiness, Cohesiveness, Springiness, and Adhesiveness. Biplots of sample scores and variable vectors were used to interpret groupings and underlying texture dimensions. Full PCA outputs, including loadings, squared cosines, and contribution values, are provided in Appendix A.

## Figures and Tables

**Table 1:** Chemical composition, energy, cholesterol adsorption capacity (CAC), and glucose adsorption capacity (GAC) of extruded high-moisture meat analogues (HMMA) with different okara flour levels (0–40%) compared with 100% defatted soy flour.

Composition/Functional Property	0% Okara	20% Okara	30% Okara	40% Okara	DSF
Moisture (%)	53.28 ± 0.44 ^a^	55.11 ± 0.26 ^b^	58.73 ± 0.29 ^c^	52.53 ± 0.47 ^a^	57.30 ± 0.43 ^b^
Protein (%)	35.06 ± 0.08 ^d^	27.25 ± 0.25 ^c^	24.56 ± 0.49 ^a^	25.75 ± 0.09 ^b^	24.63 ± 0.33 ^a^
Fat (%)	1.63 ± 0.02 ^b^	3.21 ± 0.05 ^c^	3.71 ± 0.05 ^d^	5.14 ± 0.05 ^e^	0.62 ± 0.10 ^a^
Ash (%)	1.42 ± 0.02 ^d^	1.03 ± 0.01 ^b^	1.01 ± 0.02 ^a^	1.15 ± 0.01 ^c^	3.03 ± 0.04 ^e^
Carbohydrate (%)	8.61 ± 0.51 ^a^	13.40 ± 0.56 ^c^	11.98 ± 0.37 ^b^	15.44 ± 0.47 ^e^	14.42 ± 0.68 ^d^
Total dietary fiber (g/100 g)	0.00 ± 0.00 ^a^	3.58 ± 0.02 ^c^	4.94 ± 0.04 ^d^	7.59 ± 0.08 ^e^	1.58 ± 0.02 ^b^
Total energy (kcal/100 g)	189.66 ± 2.09 ^c^	191.51 ± 0.84 ^d^	179.61 ± 1.20 ^b^	210.98 ± 2.00 ^e^	161.57 ± 1.69 ^a^
Energy from fat (kcal/100 g)	14.89 ± 0.19 ^b^	29.11 ± 0.20 ^c^	33.28 ± 0.48 ^d^	46.33 ± 0.58 ^e^	5.56 ± 0.51 ^a^
CAC (mg/g, pH 2)	4.64 ± 0.00 ^a^	4.66 ± 0.01 ^b^	4.77 ± 0.00 ^d^	4.80 ± 0.00 ^e^	4.75 ± 0.00 ^c^
CAC (mg/g, pH 7)	4.63 ± 0.00 ^a^	4.65 ± 0.00 ^b^	4.68 ± 0.00 ^c^	4.77 ± 0.00 ^e^	4.71 ± 0.00 ^d^
GAC (mM/g)	39.49 ± 2.25 ^b^	42.07 ± 1.31 ^c^	50.95 ± 1.05 ^d^	54.35 ± 2.16 ^e^	31.93 ± 1.44 ^a^

Values are mean ± SD (*n* = 3). Different superscript letters within a row indicate significant differences (*p* < 0.05). CAC: cholesterol adsorption capacity; GAC: glucose adsorption capacity.

**Table 2:** Pasting properties of high-moisture meat analogue (HMMA) flour mixtures with okara flour (0–40%) compared with 100% defatted soy flour, determined under pressurized (simulate high-moisture extrusion, 50–120 °C, 30 bar) and atmospheric (RVA-like, 50–95 °C) conditions.

Condition	Property	0% Okara	20% Okara	30% Okara	40% Okara	DSF
Pressurized	Pasting temperature (°C) ^1^	78.3 ± 0.7 ^b^	76.4 ± 0.5 ^a^	77.5 ± 0.6 ^a,b^	77.3 ± 0.8 ^a^	97.3 ± 0.7 ^c^
(50–120 °C, 30 bar)	Peak viscosity (mPa·s) ^2^	583.8 ± 0.9 ^e^	270.8 ± 10.9 ^d^	158.5 ± 1.3 ^c^	132.5 ± 1.2 ^b^	80.7 ± 1.0 ^a^
	Final viscosity (mPa·s) ^3^	228.5 ± 1.1 ^e^	201.3 ± 0.8 ^c^	104.5 ± 1.2 ^b^	210.6 ± 1.2 ^d^	50.4 ± 1.0 ^a^
Atmospheric	Pasting temperature (°C)	67.5 ± 4.5 ^b^	71.1 ± 1.8 ^c^	70.4 ± 3.6 ^c^	69.2 ± 1.9 ^c^	53.2 ± 1.5 ^a^
(50–95 °C, 1 bar)	Peak viscosity (mPa·s)	1318.3 ± 7.0 ^d^	360.7 ± 1.2 ^c^	201.5 ± 0.7 ^a^	212.5 ± 0.7 ^b^	211.4 ± 0.7 ^b^
	Final viscosity (mPa·s)	1209.7 ± 16.5 ^d^	525.9 ± 13.3 ^c^	253.3 ± 8.9 ^a^	257.1 ± 7.8 ^a^	327.6 ± 2.2 ^b^

Values are mean ± SD (*n* = 3). Different superscript letters in the same row indicate significant differences (*p* < 0.05). ^1^ Pasting temperature: temperature at which viscosity begins to rise. ^2^ Peak viscosity: maximum viscosity during heating (starch granule swelling and water uptake). ^3^ Final viscosity: viscosity after cooling, reflecting re-association/retrogradation of starch molecules.

**Table 3:** Formulation of extruded high-moisture meat analogues with varying okara flour content.

HMMA Sample	* Ratio of Flour (%)				
	Okara Flour	Soy Protein Isolate	Wheat Gluten	Corn Starch	Defatted Soy Flour
0% Okara	0	50	40	10	0
20% Okara	20	30	40	10	0
30% Okara	30	20	40	10	0
40% Okara	40	10	40	10	0
DSF	0	0	0	0	100

* Percentages are on a flour-basis (sum to 100).

## Data Availability

All related data and methods are presented in this paper. Additional inquiries should be addressed to the corresponding author.

## Supplementary Materials

The following supporting information can be downloaded at: https://www.mdpi.com/article/10.3390/gels11100805/s1, Table S1: Texture parameters of high-moisture meat analogue (HMMA) samples with varying okara incorporation (0–40%) and defatted soy flour (DSF).

## Abbreviations

The following abbreviations and variables are used in this manuscript:

**Abbreviation/Variable**	**Definition**
DSF	Defatted soy flour
GAC	Glucose adsorption capacity
HMMA	High-moisture meat analogue
HME	High-moisture extrusion
PBMA	Plant-based meat analogue
PCA	Principal component analysis
RVA	Rapid Visco Analyzer
SDS-PAGE	Sodium dodecyl sulfate–polyacrylamide gel electrophoresis
SPI	Soy protein isolate
TDF	Total dietary fiber
TPA	Texture profile analysis
p.Force	Maximum cutting force
p.Work_of_Shear	Work of shear (energy required to cut through sample)
p.Cutting_Strength	Cutting strength (force normalized by cross-sectional area)
p.Distance_at_Failure	Blade displacement at fracture
v.Force	Maximum cutting force
v.Work_of_Shear	Work of shear (energy required to cut through sample)
v.Cutting_Strength	Cutting strength (force normalized by cross-sectional area)
v.Distance_at_Failure	Blade displacement at fracture
Note: “p.” and “v.” denote directions relative to the extrusion flow, as defined in Section 4.7 (Materials and Methods).	

## Appendix A

**Table A1:** Principal component loadings, squared cosines, and contributions of the nine original cutting test variables to PC1–PC3, used for variable selection in PCA. Bolded values indicate the highest squared cosine per variable. Variables were retained based on high loading, squared cosine ≥ 0.5, and substantial contribution to PC1 or PC2.

Variable	Loading			Squared Cosine			Contribution (%)		
	PC1	PC2	PC3	PC1	PC2	PC3	PC1	PC2	PC3
Force	0.978	−0.176	0.003	**0.957**	0.031	0.000	20.504	1.229	0.002
Hardness	0.968	−0.220	0.021	**0.938**	0.049	0.000	20.092	1.921	0.107
Gumminess	0.990	−0.046	−0.049	**0.980**	0.002	0.002	20.998	0.082	0.584
Chewiness	0.992	0.025	−0.051	0.984	0.001	0.003	21.077	0.024	0.649
Cohesiveness	−0.115	0.974	−0.175	0.013	**0.948**	0.031	0.283	37.505	7.605
Resilience	0.193	0.937	−0.256	0.037	**0.878**	0.066	0.794	34.709	16.212
Springiness	0.311	0.779	0.544	0.097	**0.606**	0.296	2.076	23.982	73.308
Adhesiveness	−0.813	−0.118	0.079	**0.662**	0.014	0.006	14.174	0.547	1.533

**Table A2:** Principal component loadings, squared cosines, and contributions of the eight original TPA variables to PC1–PC3, used for variable selection in PCA. Bolded values indicate the highest squared cosine per variable. Variables were retained based on high loading, squared cosine ≥ 0.5, and substantial contribution to PC1 or PC2.

Variable Name	Loading			Squared Cosine			Contribution (%)		
	PC1	PC2	PC3	PC1	PC2	PC3	PC1	PC2	PC3
p.Force	0.932	0.323	−0.016	**0.869**	0.104	0.000	17.628	3.602	0.032
p.Work_of_Shear	0.946	−0.214	−0.073	**0.894**	0.046	0.005	18.147	1.585	0.670
p.Cutting_Strength	0.517	0.838	0.051	0.267	**0.703**	0.003	5.416	24.330	0.322
p.Distance_at_Failure	0.488	−0.768	−0.089	0.238	**0.590**	0.008	4.834	20.420	0.996
v.Force	0.986	−0.043	0.082	**0.973**	0.002	0.007	19.744	0.065	0.849
v.Work_of_Shear	0.983	−0.038	−0.009	**0.967**	0.001	0.000	19.613	0.050	0.011
v.Cutting_Strength	0.594	0.779	0.066	0.353	**0.607**	0.004	7.166	21.009	0.540
v.Distance_at_Failure	0.553	−0.728	−0.372	0.306	**0.529**	0.138	6.214	18.322	17.374
Texturization	0.247	−0.554	0.794	0.061	0.307	**0.630**	1.239	10.616	79.206
